# Supplementary material for: A music structure analysis method based on beat feature and improved residual networks
Source: PLoS One. 2025 Feb 19;20(2):e0312608. doi: 10.1371/journal.pone.0312608 (PMC11838893; doi:10.1371/journal.pone.0312608)
Supplement: Attachment — Submitted filename: Response to Reviewers.docx [file pone.0312608.s001.docx]

**Dear editor and reviewers:**

First and foremost, we extend our sincere gratitude for the thorough review of our manuscript and for the valuable suggestions and advice provided. We have carefully considered the feedback and have made corresponding revisions to the paper in light of the reviewers' recommendations. Below is our point-by-point response to the reviewers' comments:

**The first reviewer's comments:**

The article "A music structure analysis method based on beat feature fusion and improved residual networks" introduces a new approach to music structure analysis (MSA), focusing on boundary detection and segment labeling. The method refines music structure labels into nine types, segments music based on beats, and extracts various acoustic features for accurate segmentation. It employs a ResNet-34 network with a self-attentive mechanism to predict beat categories and uses post-processing to refine the results. Evaluated on the SALAMI-IA dataset, the method shows a 3% improvement over the current optimal method and outperforms others on PWF and Sf metrics, highlighting the effectiveness of combining boundary detection and segment labeling.

Here are three shortcomings of the article "A music structure analysis method based on beat feature fusion and improved residual networks":

1. Insufficient Comparative Analysis: The article lacks a thorough comparative analysis with more diverse state-of-the-art methods, limiting the context of its contributions and performance.

2. Dataset Limitations: The study only evaluates its method on the SALAMI-IA dataset, which may not fully represent the method's robustness and generalizability across different musical genres and datasets.

3. Complexity of the Model: The proposed method, which includes DANet+ResNet-34 and beat feature fusion, might be too complex for practical applications, and the article does not discuss the computational costs or efficiency of the approach.

**Reply 1**：Thank you for the reviewer’s constructive feedback suggesting that the article lacks a thorough comparative analysis with more diverse state-of-the-art methods. We deeply appreciate the reviewer’s vigilance and the opportunity to clarify and correct this issue. We acknowledge that our paper does lack in-depth comparative analysis with advanced methods from different fields. Due to the initial strategy selection in the research, we only selected some music structure analysis methods for horizontal comparative analysis with the methods proposed in this paper in section 3.1.5. To address the issue of limitations on the contribution and performance of the paper due to the lack of comparative analysis, we will provide a detailed explanation of the horizontal comparative experiments in this paper to highlight their context of contributions and performance. The revised content is in paragraph 1 of section 3.1.5.

The additions are as follows:

In order to the comparison of the contribution of the models on boundary detection and Segment labeling, some MSA methods are selected for comparison with the methods in this paper. Scluster (a) is a classical approach to clustering. Scluster is capable of handling large-scale datasets due to its relatively low computational complexity. SpecTNT (f) is a multi-point method using spectral-temporal converter + CTL loss-based. SpecTNT demonstrates advanced performance in music tagging and vocal melody extraction. Supervised CNN (b) is a convolutional neural network method for two-stage classification. It plays an important role in multiple fields such as image recognition and object detection. LSTM-HSMM (c) is a hybrid model of hidden semi-Markov model and recurrent neural network. LSTM-HSMM not only offers a profound understanding of sequential data but also facilitates precise prediction and classification of the underlying states within the data.Non-negative Tucker decomposition (NTD) ‎[12] (d) is a non-negative Tucker decomposition. DSF+Scluster‎[13] (e) is a metric learning method after a supervised approach. This method has significant advantages in fine-grained recognition at the individual level.

**Reply 2**：Thank you for the reviewer’s observation regarding the study only evaluates its method on the SALAMI-IA dataset, which may not fully represent the method's robustness and generalizability across different musical genres and datasets.

We acknowledge that our study only evaluates its method on the SALAMI-IA dataset. Due to the incomplete introduction of this dataset in this paper, readers may feel that it may not fully represent the method's robustness and generalizability across different musical genres and datasets. This dataset actually contains different types of music, which is sufficient to support the relevant experiments in this paper. Moreover, this dataset is open source. The revised content is in paragraph 1 of section 2.2.1.

The additions are as follows:

A subset of the Internet of the SALAMI public dataset ‎[30] (Called SALAMI-IA) was used in the experiments of this paper. SALAMI-IA is a database containing a large number of popular, jazz, classical and world music genres. The SALAMI-IA dataset is characterized by its annotated music hierarchy (Lead instrument track, Function track, and music similarity track, respectively). Meanwhile, the size and content of SALAMI-IA dataset is publicly available and downloadable. Therefore, it is used as part of the evaluation dataset by many MIREX music structure segmentation competitions.

**Reply 3**：In response to the reviewer's question: The proposed method, which includes DANet+ResNet-34 and beat feature fusion, might be too complex for practical applications, and the article does not discuss the computational costs or efficiency of the approach. We provide the following explanation: As shown in Table 10 of the horizontal comparison experiment in section 3.2.4, the DANet+ResNet-34 method proposed in this paper has an average accuracy in analyzing the music structure of various genres in the SALAMI genres dataset. Compared with other methods, it has obvious advantages, which proves the universality and superiority of the music structure analysis method proposed in this paper. This is attributed to the fact that the method proposed in this paper can not only analyze the structure of music from different genres, but also improve the accuracy of music structure analysis. Regarding the lack of discussion on computational cost or efficiency in this paper, we did consider this issue in the early stages of the research. Finally, considering factors such as data sample size, model parameter size, and hardware conditions, we decided to use this method for relevant experiments. We will add relevant discussion explanations to the introduction section. The revised content is in paragraph 4 of introduction.

The additions are as follows:

In this paper, in response to the issues of insufficient audio feature representation and insufficient model generalization ability in music structure analysis methods, a complete MSA processing system is designed to accomplish the above two tasks sequentially. Taking into account factors such as the sample size of the dataset used in this paper, the size of the model parameters involved, and hardware conditions, we have decided to use this method for relevant research.

**The second reviewer's comments:**

This paper proposes simultaneous boundary detection and segment labeling based on beat feature fusion. This paper is adequately interesting. However, the crucial problems and proposed solutions are not well explained in the introduction or abstract. The statement, "The accuracy of segment labeling will be affected by the accuracy of boundary detection, and the two are inseparable," requires confirmation. The author explains that existing methods utilize a fixed time length for frame segmentation in the time-frequency domain, whereas this work segments frames based on beats (introduction: 4^th^ paragraph, second sentence). However, the subsections on beat division and data processing describe using fixed window lengths of 32 ms and 1024 samples, respectively. This raises the question of how the proposed method explains frame segmentation based on beats. The author needs to describe frame segmentation based on beats more clearly. I have outlined some concerns that require responses as

follows:

1. Please reconsider the title of this paper, as it is too general and not specific. The core objective of this research is the classification of beat categories.

2. In the introduction, the author argues that the accuracy of segment labeling will affect the accuracy of boundary detection, and the two cannot be separated (3rd sentence in the 3^rd^ paragraph). This argument needs to be supported by facts. The previous research described by the author briefly explains the methods used but does not support the stated argument.

3. The problem and proposed solution should be explained in a structured manner in the introduction. What is the crucial problem?

4. Abstract, the author does not emphasize the crucial issue of why boundary segmentation and music labeling based on beats need to be done simultaneously

5. The word “And” cannot be used at the beginning of a sentence.

6. The part explaining boundary detection in Fig.1 required to be visualized.

7. The caption of Fig.1 should be adjusted to the objective of the proposed method.

8. In the last paragraph of the introduction section, 'In this paper, a complete MSA processing system is designed to accomplish the above two tasks simultaneously.' Be cautious with the term 'simultaneously,' as it implies that the proposed method is end-to-end, meaning it can produce outputs for both tasks at the same time. However, the framework shown in Fig. 1 is sequential: The first step performs boundary detection based on beat feature fusion and then uses the output for segment labeling. Therefore, the term 'simultaneously' is not suitable to describe your statement."

9. In the abstract and introduction, the author introduces 'boundary detection' as one of the tasks to be addressed. However, boundary detection is not described in the method section. All terms mentioned should be clearly described in the method section

10. Preferably section 2 should be Materials and Methods. The dataset can be written in this section.Is the Data Processing stage related to feature extraction? If yes, combine both subsections.

11. In the titile autor mention ‘beat feature fusion’, however this term is not explained in the method.

12. This paper proposes an improved ResNet-34 by incorporating a lightweight DANet model for beat category classification. Could you clarify the relevance of ResNet-34 and DANet to your data? For example, beat detection involves extracting temporal features, therefore, Temporal Convolutional Network (TCN) is used as proposed model to capture the sequential feature in the music structure.

13. The caption of Figure 4 should be adjusted to match the proposed model name

**Reply 1**：Thank you for the reviewer's suggestion that the title of this paper is too general and not specific. The suggestion has important guiding significance for our paper. We have carefully considered your suggestions on the title of the paper and believe that it is necessary to reflect the core content of our research more specifically and accurately. We deeply appreciate the reviewer’s vigilance and the opportunity to clarify and correct this issue. We acknowledge that the original title may have been too broad and failed to clearly convey the main focus of this study. After careful consideration, we have decided to change the title " A music structure analysis method based on beat feature fusion and improved residual networks" to " A music structure analysis method based on beat feature and improved residual networks". In order to more accurately reflect the innovative points and goals of our research. We believe that this new title not only attracts the research interest of peers, but also helps potential readers quickly grasp the main idea of the article. During the revision process, we ensured that other parts of the text were also adjusted accordingly to ensure consistency and coherence throughout the entire article. We expect these improvements to meet the standards of the journal and bring value to the academic community.

**Reply 2**：Thank you for the reviewer’s insightful question about the argument that the accuracy of segment labeling will affect the accuracy of boundary detection and the two cannot be separated needs to be supported by facts. Reviewer's query has provided us with an opportunity to enhance our exposition on this topic.

We acknowledge that our initial submission did not support the argument through facts. After examining the context of the paper, we have decided to delete this paragraph, which will not affect the structure of the paper.

**Reply 3**：Thank you to the reviewer for reminding us that the problem and proposed solution should be explained in a structured manner in the introduction. The suggestion that the questions and solutions you raised should be explained in a structured manner in the introduction is very valuable, as it will help improve the logic and clarity of the paper. We acknowledge that our initial submission focused primarily on the conceptual approach and the display of experimental results. Inadvertently overlooking the importance of the raising of the problem in the introduction.Upon careful consideration of your feedback, we have decided to include the missing problem in the introduction to ensure completeness and clarity. We believe that through these adjustments, our introduction will more effectively provide readers with the background, questions, objectives, and expected results of the research, while maintaining the compactness and attractiveness of the introduction.The revised content is in the introduction.

The additions are as follows:

In this paper, in response to the issues of insufficient audio feature representation and insufficient model generalization ability in music structure analysis methods, a complete MSA processing system is designed to accomplish the above two tasks sequentially. Taking into account factors such as the sample size of the dataset used in this paper, the size of the model parameters involved, and hardware conditions, we have decided to use this method for relevant research.

**Reply 4**：Thank you to the reviewer's careful review and valuable feedback. We deeply appreciate the reviewer’s vigilance and the opportunity to clarify and correct this issue. In response to the reviewer's question: The author does not emphasize the crucial issue of why boundary segmentation and music labeling based on beats need to be done simultaneously in abstract.

In the eighth suggestion given by the reviewer, we understand that these two tasks are not carried out simultaneously, so we have indicated in our response to the eighth suggestion that we will modify this type of description. Music structure analysis (MSA) contains two tasks, boundary detection and segment labeling. Boundary detection and Segment labeling are expected to accurately divide music segments and clarify the function. In this paper, a method is studied to accomplish the two tasks. It can be seen from Figure 1 of this paper. the framework is sequential: The first step performs boundary detection based on beat feature fusion and then uses the output for segment labeling. Finally, a smooth filtering post-processing method was used to correct the classification results, improving the accuracy of music structure analysis. Therefore, we should not emphasize in the abstract that these two tasks need to be carried out simultaneously.

**Reply 5**：Thank you to the reviewer's careful review and valuable feedback. We sincerely apologize for the grammar error you pointed out about A: The word “And” cannot be used at the beginning of a sentence. Through reading relevant literature and searching for information, we found that 'and' is a rhetorical device used at the beginning to emphasize and emphasize, often used in conversations or speeches. The term 'And' is not suitable for emotionless articles that primarily provide information, as it may appear abrupt in such contexts. We have checked the entire text and resolved this formatting issue.

The modified content is as follows:

The various methods mentioned above generally start segment labeling after estimating the boundary. Segment labeling must be done based on boundary detection. It has inspired many scholars to try to combine the boundary detection and segment labeling tasks.

Finally, all beat samples are divided into three parts, which are the training set, validation set and test set. The total number of beat samples in the training set, validation set and test set are 191436, 47860 and 59824, as shown in Table 1.

Therefore, in order to make a comprehensive consideration for MSA research, 14 time-frequency features that reflect musicality are designed in this paper. The time-domain features include tonal mass features Tonnetz for pitch analysis; the Chroma Energy Normalized Statistics (CENS) that calculates the audio time domain energy; The rhythmic features include Tempogram, which represents the rhythm represented on a time scale.

**Reply 6**：Thank you to the reviewer for reminding us that the part explaining boundary detection in Fig.1 required to be visualized.

After careful examination, we found that there is indeed a lack of explanation for boundary detection in Fig.1. The first step performs boundary detection based on beat feature fusion and then uses the output for segment labeling. So boundary detection should be between the fusion of beat features and segment labeling. The revised content is in the Figure 1 of section 2.1. We believe that through these supplements and improvements, the boundary detection section of Figure 1 can now provide readers with a clearer and more comprehensive understanding.

The modified image is as follows:

Fig. 1. The system framework of Music Structure Analysis.

**Reply 7**：Thank you for the reviewer's suggestion that the caption of Fig.1 should be adjusted to the objective of the proposed method. We deeply appreciate the reviewer’s vigilance and the opportunity to clarify and correct this issue.Based on the suggestions made by the reviewers, we have realized that the caption of Figure 1 is indeed inappropriate. The system framework of the music structure analysis method based on beat feature fusion and residual network shown in Figure 1 mainly consists of three steps: beat feature fusion, boundary detection and paragraph labeling, and label post-processing. The fusion of beat features completes the music data preprocessing for music structure analysis methods. Boundary detection and paragraph labeling are responsible for predicting music and obtaining the structure of music paragraphs. The post-processing step involves processing the predicted labels to further improve accuracy. Figure 1 shows the objective of the proposed method. So we have made changes to the caption of Figure 1. The revised content is in the caption of Figure 1 of section 2.1.

The revised content is as follows:

Fig. 1. The system framework of Music Structure Analysis.

**Reply 8**：Thank you for bringing to our attention the inconsistency on page 7 of our manuscript. We deeply appreciate the reviewer’s vigilance and the opportunity to clarify and correct this issue.

We fully agree with your opinion that the framework shown in Figure 1 is indeed executed sequentially, rather than simultaneously. We recognize that this statement may cause misunderstandings among readers, so we have made corresponding modifications to the introduction section. Music structure analysis (MSA) contains two tasks, boundary detection and segment labeling. Boundary detection and Segment labeling are expected to accurately divide music segments and clarify the function. In this paper, a method is studied to accomplish the two tasks. It can be seen from Figure 1 of this paper. the framework is sequential: The first step performs boundary detection based on beat feature fusion and then uses the output for segment labeling. Finally, a smooth filtering post-processing method was used to correct the classification results, improving the accuracy of music structure analysis. To address this, we have revised the text to ensure that the description is accurate and free from ambiguity. This statement more accurately describes the actual operational process of our method. The revised content is in the last paragraph of the introduction section.

The revised content is as follows:

In this paper, in response to the issues of insufficient audio feature representation and insufficient model generalization ability in music structure analysis methods, a complete MSA processing system is designed to accomplish the above two tasks sequentially. Taking into account factors such as the sample size of the dataset used in this paper, the size of the model parameters involved, and hardware conditions, we have decided to use this method for relevant research.

**Reply 9**：Thank you to the reviewer for reminding us that boundary detection is not described in the method section. We appreciate the opportunity to address these points more thoroughly. We acknowledge that we did not provide a clear description of the term boundary detection in the methodology section. As one of the tasks that needs to be addressed, the importance of boundary detection is self-evident. In this paper, the first step is to perform boundary detection based on beat feature fusion, and then use the output for labeling. Therefore, after introducing the beat feature fusion stage, we will provide a detailed introduction to boundary detection. The revised content is in paragraph 1 of section 2.4.

The revised content is as follows:

After the feature extraction for each small segment is completed, it needs to be perform boundary detection and fed into a classification network for category labeling. Boundary detection in music structure analysis is a crucial step that involves identifying and locating the starting and ending points of different parts in a musical composition. Boundary detection helps with further analysis tasks such as identifying music structures, classifying music styles, and retrieving music information. Boundary detection is usually achieved by analyzing the characteristics of music signals. These features may include timbre, rhythm, pitch, and other musical attributes. The selection of boundary detection algorithms is crucial for the accuracy of the results. Different algorithms may have different recognition effects on music structures, and some algorithms may be more sensitive to boundary detection of certain music styles. In addition, the performance of boundary detection also depends on the quality of the extracted music features and the parameter settings of the algorithm. Some research works attempt to improve the accuracy and robustness of boundary detection by combining multiple features and algorithms.

**Reply 10**：Thank you for the reviewer's suggestion that preferably section 2 should be Materials and Methods. We deeply appreciate the reviewer’s vigilance and the opportunity to clarify and correct this issue.

We acknowledge that the data processing stage is related to feature extraction. Based on your suggestion, we have reorganized Section 2 to ensure clarity and coherence of materials and methods. We have taken special note of your guidance on datasets and feature extraction stages. During the feature extraction process, a total of 14 acoustic features were extracted in both time and frequency domains for each beat. This includes both time-domain and frequency-domain features, ensuring that each beat will have a corresponding feature vector. We transfer the introduction and processing of the dataset to the second section of this paper, and change the title of the second section to Materials and Methods. We believe that through these modifications, Section 2 now closely follows the standard structure of materials and methods, while also presenting the correlation between the dataset and feature extraction in more detail.

**Reply 11**：Thank you for the reviewer's suggestion that we did not introduce the term 'beat feature fusion' when introducing the beat feature fusion method. We have decided to include a missing introduction to the term 'beat feature fusion' in the manuscript to ensure completeness and clarity. Although we have made changes to the title in reply 1, the term 'beat feature fusion' is still part of the structure of this paper and it is still necessary to explain this term. The music structure analysis method based on beat feature fusion and residual network designed in this paper consists of three main steps: beat feature fusion, paragraph labeling, and label post-processing. The step of special fusion of beats is to process the original music signal through beat segmentation, feature extraction, and feature fusion in sequence, which is the foundation of the entire music analysis. Therefore, we will introduce the fusion of beat features in the final step of this stage. The revised content can be found in section 2.3.3, paragraph 1.

The additions are as follows:

After completing the beat division and feature extraction, each beat is fused with features to form a feature fusion, which is then fed into the neural network model, in which a sliding window is used to catch temporal context information. The fusion of beat features refers to combining these beat related features with other acoustic or musical features to obtain a more comprehensive description of the music signal. In music structure analysis, the fusion of beat features is a key process that involves combining different beat features to obtain a more comprehensive and accurate representation of music rhythm. These features may include the intensity, speed, duration, etc. of rhythm, which are important semantic features reflecting different genres and styles of music. The fusion of beat features also plays an important role in music genre classification. By combining beat semantics and MFCC acoustic features, the accuracy of music genre classification can be improved. This indicates that feature fusion not only helps improve the detection and classification of music rhythms, but also enhances the performance of music genre recognition. A sliding window covers four beats, i.e., the feature matrix of beat i is the concatenation of feature vectors of beats i, i-1, i-2 and i+1. This input method enables the model to learn different segments of different songs at the same time.

**Reply 12**：Thank you to the reviewer's careful review and valuable feedback. We deeply appreciate the reviewer’s vigilance and the opportunity to clarify and correct this issue. In response to the reviewer's question: Could you clarify the relevance of ResNet-34 and DANet to your data? Our explanation is as follows:

ResNet-34 is a deep residual network proposed by Microsoft Labs, known for its deep network structure and residual module, which effectively solves the problem of gradient vanishing or exploding in traditional convolutional neural networks when deepening the network layers. ResNet-34, as a powerful image recognition model, can enhance its feature extraction ability by integrating lightweight DANet models, which may improve the performance of beat category classification tasks on the SALAMI-IA dataset. The lightweight DANet model is an attention mechanism network that enhances the model's feature extraction ability by aggregating spatial features and contextual information. Integrating DANet into ResNet-34 can further improve the performance of the model for beat category classification tasks, especially when processing music or sound signal related data.

**Reply 13**：Thank you for the reviewer's suggestion that the caption of Figure 4 should be adjusted to match the proposed model name. We deeply appreciate the reviewer’s vigilance and the opportunity to clarify and correct this issue.Based on the suggestions made by the reviewers, we have realized that the caption of Figure 4 is indeed inappropriate. In order to maintain the rigor and clarity of the paper, all key terms and tasks mentioned should have clear definitions and descriptions in the methodology section. The content shown in Figure 4 is actually the complete structure of the model proposed in this paper, which consists of convolutional and BN layers, four similarity layers, DANet module, pooling layer, and fully connected layer. The caption of Figure 4 as Structure diagram of the proposed model is unreasonable and should be adjusted to Residual network structure based on the attention mechanism DANet. So we have made changes to the caption of Figure 4. We believe that through these supplements and modifications, the methodology section of the paper can now clearly describe the relevant terminology and technical details of boundary detection, meeting the requirements of academic rigor. The revised content is in the caption of Figure 4 of section 2.4.

The revised content is as follows:

Fig. 4. Residual network structure based on the attention mechanism DANet.

Comprehensive Review of Formatting: Taking your observations to heart, we have meticulously reviewed all graphics and tables in the article. We have made the necessary corrections to ensure that they are all in compliance with the journal's formatting standards

Firstly, we would like to express our sincere gratitude for the valuable opinions and suggestions you have provided. Your profound insights and professional opinions have immeasurable value for our research work. After carefully considering your feedback, we have made a comprehensive revision to the manuscript. This includes improvements in data and methodology, clarification of theoretical frameworks, in-depth discussions of results, and polishing of language and format. We believe that with these revisions, our research work will become more comprehensive and provide valuable insights for the academic community. Thank you again for your valuable time and professional review. We look forward to your further feedback and hope that our revisions will meet the standards of the journal.

Thank you once again for your thoughtful review and constructive feedback.

Warm regards,

Yi Guo
